# Air pollutants and ovarian reserve: a systematic review of the evidence

**DOI:** 10.3389/fpubh.2024.1425876

**Published:** 2024-09-23

**Authors:** Roberta Zupo, Fabio Castellana, Tim S. Nawrot, Luisa Lampignano, Ilaria Bortone, Ferdinando Murgia, Gianluca Campobasso, Agnieskza Gruszecka Kosowska, Orazio Valerio Giannico, Rodolfo Sardone

**Affiliations:** ^1^Department of Interdisciplinary Medicine (DIM), University of Bari Aldo Moro, Piazza Giulio Cesare, Bari, Italy; ^2^Centre for Environmental Sciences, Hasselt University, Diepenbeek, Belgium; ^3^Department of Public Health and Primary Care, Environment and Health Unit, Leuven University, Leuven, Belgium; ^4^Local Healthcare Authority of Bari, ASL Bari, Bari, Italy; ^5^Department of Translational Biomedicine and Neuroscience "DiBraiN", University of Bari "Aldo Moro", Bari, Italy; ^6^Department of Obstetrics and Gynecology, "Miulli" General Hospital, Bari, Italy; ^7^Obstetrics and Gynecology Unit, Vito Fazzi Hospital, Lecce, Italy; ^8^Department of Environmental Protection, Faculty of Geology, Geophysics and Environmental Protection, AGH University of Krakow, Al. Mickiewicza, Krakow, Poland; ^9^Unit of Statistics and Epidemiology, Local Health Authority of Taranto, Taranto, Italy; ^10^Department of Eye and Vision Sciences, University of Liverpool, Liverpool, United Kingdom

**Keywords:** air pollutants, fine particulate matter, ovarian reserve, fertility, systematic review

## Abstract

**Background:**

Growing evidence indicates an association between ambient air pollution and decreased human reproductive potential. This study aims to systematically review the association between air pollutants and female ovarian reserve.

**Methods:**

The literature was searched in six electronic databases through June 2024. Screening the 136 articles retrieved for inclusion criteria resulted in the selection of 15 human observational studies that evaluated the effect of environmental pollutants on ovarian reserve markers. The study protocol was registered on the International Prospective Register of Systematic Reviews (PROSPERO, registration code: CRD42023474218).

**Results:**

The study design of the selected studies was found to be cross-sectional (2 of 15), retrospective cohort (10 of 15), prospective cohort (2 of 15), and case–control (1 of 15). The study population was distributed as follows: Asians (53%, eight studies), Americans (33%, five studies), and Europeans (14%, two studies). The main findings showed a higher body of evidence for the environmental pollutants PM2.5, PM10, and NO_2_, while a low body of evidence for PM1, O_3_, SO_2_, and a very low body of evidence for benzene, formaldehyde, and benzo(a)pyrene, yet consistently showing significant inverse association data. The overall methodological quality of the selected studies was rated moderated across the 14 domains of the National Institutes of Health (NIH) toolkit.

**Conclusion:**

The data suggest that increased exposure to air pollutants seems to be associated with reduced ovarian reserve, with the most substantial evidence for pollutants such as PM2.5, PM10, and NO_2_. However, more evidence is needed to draw conclusions about causality.

## Introduction

Public health data on air pollution from the global burden of disease (GBD) estimates 213 million disability-adjusted life years (DALYs)—equal to 0.84% of the global DALY—6.67 million deaths in 2019 (1). Much of the scientific community has supported the association between air pollution and the risk of cardiovascular (2–4), respiratory (5, 6), endocrine (7), reproductive (8), and all-cause mortality to date (9, 10). Until now, there is substantial consistency around biological mechanisms involving inflammation, oxidative stress, endocrine disruption, and epigenetic changes.

Some early studies pointed to air pollution exposure being associated with reduced fertility and a range of adverse pregnancy outcomes, such as miscarriage, preterm delivery, and stillbirth, regardless of having natural pregnancies or undergoing assisted reproductive technologies (2, 11–14). The underlying mechanism of female fertility decline due to air pollutants remains unclear. At the same time, limited evidence speculates that impaired ovarian reserve caused by oxidative stress and inflammatory response caused by air pollution May be a critical path.

The number and quality of the ovarian follicle pool are commonly referred to as ovarian reserve, which indicates a woman’s reproductive potential or fertility (15). After puberty, follicle development begins under gonadotropin stimulation (16), and the entire development process mainly includes the development of a small number of primordial follicles to the antral stage and the selection of an antral follicle for growth to the preovulatory stage during each menstrual cycle (16).

In clinical settings, a common practice is to use hormonal and ultrasound markers as proxies of ovarian reserve (17, 18). In this context, ultrasound antral follicle count (AFC), serum levels of follicle-stimulating hormone (FSH), anti-Müllerian hormone (AMH), inhibin B, and E2 have been proposed as potential markers of fertility, among which AMH is considered the most sensitive and specific available marker (19, 20). Indeed, AFC results tend to have operator skill-dependent variability, whereas serum AMH is the best predictor of ovarian reserve for its high representativeness of small AFC (19, 20). Also, previous studies found that AMH levels remain stable during the menstrual cycle and can be detected on any day of the period (21).

Animal studies have documented that exposure to particulate matter 2.5 (PM2.5) is associated with decreased levels of reproductive hormones and the number of antral and primordial ovarian follicles in mice. Gai and colleagues showed that PM2.5 reduced AMH levels and increased interleukin 6 (IL-6) and tumor necrosis factor-alpha (TNF-*α*) levels in mouse ovarian tissue (22). A significant reduction in the proportion of primordial follicles was observed by Ogliari and colleagues in mice exposed to diesel exhaust with doses equal to the average daily levels of PM2.5 (fine particles in ambient air 2.5 μm or less in size) reported by the World Health Organization (23).

However, till today, the body of evidence on the association between exposure to air pollutants and markers of ovarian reserve in women lacks a synthesis of evidence. Therefore, to fill this gap, this study aimed to systematically investigate the association between major environmental air pollutants and female fertility in childbearing females.

## Methods

### Search strategy, study selection, and data extraction

A computer search of the literature on databases, namely, MEDLINE and the Cochrane Library, identified no previous systematic reviews on exposure to environmental pollutants and ovarian reserve in women. The present systematic review followed the Preferred Reporting Items for Systematic Reviews and Meta-Analyses (PRISMA) guidelines, adhering to the PRISMA 27-item checklist (Page et al., 2021). An *a priori* protocol for search strategy and inclusion criteria was established and registered, with no particular changes to the information provided at the time of registration on the International Prospective Register of Systematic Reviews (PROSPERO), an international prospective registry of systematic reviews (CRD42023474218). We performed separate searches in the US National Library of Medicine (PubMed), Medical Literature Analysis and Retrieval System Online (MEDLINE), EMBASE, Scopus, Ovid, and Google Scholar to find human observational studies that evaluated the effect of major environmental pollutants [PM1, PM2.5, PM10, NO_2_, O_3_, SO_2_, CO, polycyclic aromatic hydrocarbons (PAHs), black carbon, 1,3-butadiene, benzene, diesel PM, formaldehyde, methylene chloride, and tetrachloroethylene] on female fertility expressed by recognized markers such as AMH, poor ovarian reserve (POR), and antral follicle count (AFC). Therefore, the primary objective was to assess the amount, consistency, and direction of the association between any of these pollutants and markers of ovarian reserve. We also considered the gray literature using the vast archive of preprints^1^ the study selection phase and the database^2^ to access abstracts of significant conferences and other unreviewed material.

The following criteria were applied to include various studies in the analysis: (1) human observational study; (2) reporting of the effect of environmental pollutants on women’s fertility as expressed by recognized ovarian reserve markers; and (3) studies that involved women of childbearing age. Animal studies, conference abstracts, reviews, letters, editorials, nonclinical trial studies, and studies involving children and/or adolescents were excluded.

The search strategy used in PubMed and MEDLINE and adapted to the other four electronic sources included keywords such as antral follicle count, ovarian reserve, PM, black carbon, and air pollutant(s) combined through the use of Boolean indicators such as AND and OR (Table 1). The search strategy used the Boolean indicator NOT to exclude opinion articles, letters, reviews, and meta-analyses. The literature search had no time restrictions, and articles were retrieved until June 2024. Two researchers (RZ and FC) searched the articles—separately and in duplicate—reviewed the titles and abstracts of the retrieved articles, checked the full texts, and selected the articles for inclusion in the study. Interrater reliability (IRR) was used to estimate intercoder agreement and then *κ* statistic to measure accuracy and precision. According to PRISMA concepts and quality assessment steps, a κ coefficient of at least 0.9 was obtained in all data extraction steps (24).

**Table 1:** Search strategy used in the US national library of medicine (PubMed) and medical literature analysis and retrieval system online (MEDLINE) and adapted to the other sources, according to selected descriptors.

	Strategy	Descriptors used
#1	Population	(Women[tiab]) OR (female*[tiab]) OR (childbearing[tiab]) OR (fertil*[tiab])
#2	Intervention/Exposure	(Particulate matter[tiab]) OR (black carbo[tiab]) OR (air pollut*[tiab]) OR (ambient pollut*[tiab])
#3	Comparator	(Categor*[tiab]) OR (exposure[tiab]) OR (occupational exposure[tiab]) OR (tertile*[tiab]) OR (quartile*[tiab]) OR (quintile*[tiab]) OR (level*[tiab])
#4	Outcomes	(Antral follicle count[tiab]) OR (oocyt*[tiab]) OR (Follicle-stimulating hormone*[tiab]) OR (Ovarian Reserve[tiab])
#5	*Exclusion keywords*	(Review[tiab]) OR (systematic review[tiab]) OR (narrative review[tiab]) OR (meta-analysis[tiab]) OR (editorial[tiab]) OR (letter[tiab]) OR (commentary[tiab]) OR (perspective[tiab]) OR (book[tiab])
#6	*Search strategy*	#1 AND #2 AND #3 AND #4 NOT #5
	Filters: Sort by: Most Recent. Date: 31 June 2024. Time restriction: none.	

### Quality assessment within and across studies and overall quality assessment

The methodological quality of the included studies was independently assessed by two researchers (RZ and FC) using the National Institutes of Health Quality Assessment Toolkits for Observational Cohort and Cross-Sectional Studies (25, 26). According to the criteria given in the toolkit, the ratings—high (good), fair (moderate), or poor—were assigned to the studies. This toolkit contains 14 questions assessing several aspects associated with the risk of bias, types I and II errors, transparency, and confounding factors: study question, population, participation rate, inclusion criteria, sample size justification, time of exposure/outcome measurement, timing, exposure levels, defined exposure, blinded assessors, repeated exposure, defined outcomes, loss to follow-up, and confounding factors. Items 6, 7, and 13 did not refer to cross-sectional studies; the maximum possible scores for cross-sectional and prospective studies were 8 and 14, respectively. Disagreements on the methodological quality of the included studies (e.g., interpretation of toolkit domains, appropriateness, and response type) between the two investigators were resolved through discussion until a consensus was reached with a third investigator (RS). A modified version of the grading system, namely, Grading of Recommendations Assessment, Development, and Evaluation (GRADE) (27) was used to assess the quality of evidence of the studies included in this systematic review. The following factors were considered: strength of association between air pollutants exposure and related female fertility, methodological quality/study design, consistency, bias, precision, size, and (where possible) dose–response gradient of effect estimates in the evidence base. Evidence was graded as very low, low, moderate, and high, as in the GRADE grading system.

## Results

**Figure 1:**
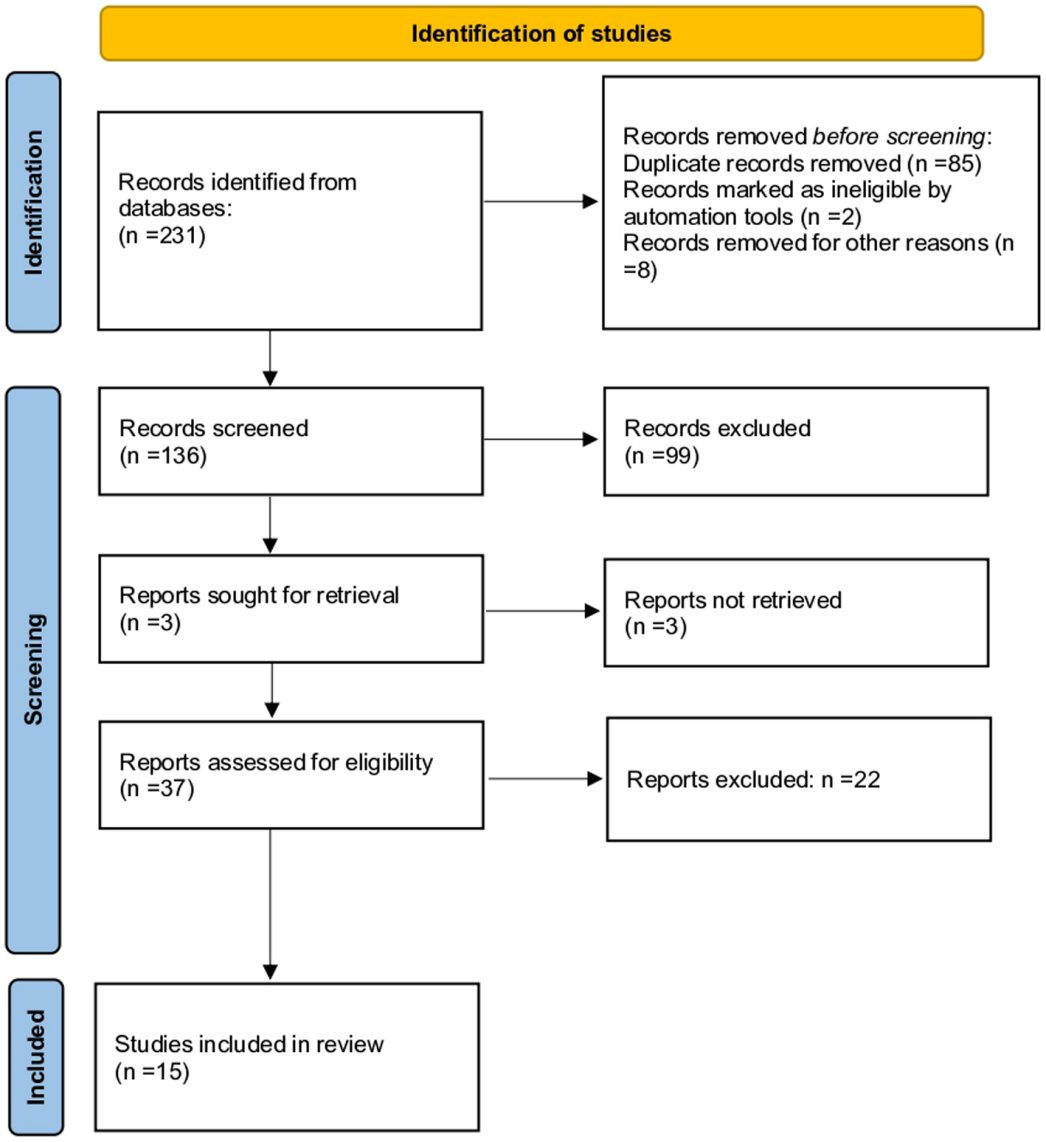
The Preferred Reporting Items for Systematic Reviews and Meta-analyses (PRISMA) flow chart illustrating the number of studies at each stage of the review.

The first systematic search of the literature yielded 321 entries. After excluding the duplicates, 136 were classified as potentially relevant and selected for the title and abstract analysis. Then, 99 were excluded for not meeting the characteristics of the approach or the review goal. After reviewing the full text of the remaining records, only 15 met the inclusion criteria and were included in the systematic review (28–42). The PRISMA flowchart illustrating the number of studies at each stage of the review is shown in Figure 1. The final study base included 15 observational studies reporting on the effect of environmental pollutants on markers of ovarian reserve in childbearing females.

The details of the study design, sample size (*N*), country, author(s) and year of publication, exclusion criteria, population age, exposure pollutant(s), outcome(s) of ovarian reserve, significant findings, and covariates considered for adjust models are provided in Table 2.

**Table 2:** Description of selected studies exploring the association between air pollutants and markers of ovarian reserve in childbearing women, *N* = 12.

Author, Year	Design	Country	*N*	Exclusion criteria	Age (years)	Exposure	Outcome	Main findings	Covariates
Wieczorek K, 2024	Retrospective	Europe	511	Women with three spontaneous miscarriages, more than three *in vitro* fertilization procedures, chemotherapy or radiotherapy of the pelvis, premature ovarian failure, previous surgical treatment of the ovaries, polycystic ovary syndrome, cyst in the ovaries with endometrium, hyperprolactinemia, hypogonadotropic and hypogonadism were excluded.		PM2.5 SO_2_	AMH AFC	A negative association between exposure to PM2.5 and AMH levels and AFC was found. Additionally, exposure to SO_2_ in the fourth quartile of exposure compared to the first one decreases the AFC.	BMI (kg/m^2^), age (years), smoking (no/yes), initial infertility diagnosis (male factor; female factor; unexplained), and the second model for age, BMI, smoking, infertility diagnosis, duration of infertility (1–3 years; 3–5 years; > 5 years); alcohol consumption (none or < 1 drink/week; 1–3 drinks/week; everyday).
Liu S, 2024	Retrospective	Asia	4,544	Diagnosis of polycystic ovary syndrome, chromosomal abnormalities, hyperprolactinemia, and women younger than 20 years or older than 49 years or with missing AMH information.	20–49	O_3_	AMH	Significantly increased risk of decreased ovarian reserve occurred with decreased AMH levels associated with environmental O_3_ exposure in Chinese women of reproductive age, especially during the secondary antral follicle stage and 1 year before measurement.	BMI, ethnicity, education, current working status, lifestyle (current smoking status), reproductive factors (infertility factors, parity, regular menstrual cycle, cycle types, and season of AMH measurement).
Liu S, 2023	Retrospective	Asia	5,189	Participants with polycystic ovary syndrome, chromosomal abnormality, hyperprolactinemia, hypothyroidism, history of ovariectomy, age < 20 or > 49 years, and incomplete residency information.	20–49	PM2.5	AMH	Significant decrease in AMH levels and increased risk of low AMH associated with PM2.5 exposure during the secondary and antral phase and 1 year before the measurement, even below the current Chinese air quality standard on PM2.5 concentrations (75 μg/m^3^).	Age, BMI, education, current smoking status, employment status, residence, the duration of infertility, infertility factors, parity, and season at AMH measurement based on previous publications
Pang L, 2023	Retrospective	Asia	18,878	Incomplete medical information and exposure data, abnormal chromosomal karyotype, PCOS, ovaries surgical treatment, chemotherapy or radiotherapy, hyperprolactinemia, hypothyroidism, or immunologic diseases under RPL at least 3 times.	20–50	PM2.5 PM10 NO_2_ O_3_	AMH	Higher exposure to PM1, PM2.5, PM10 and NO_2_ were significantly associated with a substantial decline in AMH.	Age, BMI, city, educational level, pattern of menstrual cycles, parity, duration of infertility, season of AMH, calendar year of AMH measurement, short-term air pollution, short-term temperature, and short-term relative humidity.
Li H, 2023	Retrospective	America	2,447		25–42	1,3-Butadiene Benzene Diesel particulate matter Formaldehyde Methylene chloride Tetrachloroethylene	AMH	Single-exposure models showed negative associations of AMH with benzene and formaldehyde.	
Wu S, 2022	Retrospective	Asia	2,186	Women who underwent natural cycle or mild stimulation regimen, >40 years of age, used preimplantation genetic testing, used donor oocyte or sperm, had missing residential address, and had a history of ovarian surgery.	<40	PM2.5 PM10 O_3_ NO_2_ CO SO_2_	POR	There is a positive association between exposure to SO_2_ and the risk of POR, especially for women aged 30 years and unexpected POR. Moreover, women with unexpected POR and poor responders had a lower level of long-term exposure to O_3_, compared with the matched women with NOR.	Age, BMI, smoking status, infertility cause, COH protocol, starting dose of Gn, AMH, FSH, LH, E2, and residential city.
Gregoire AM, 2021	Cross-sectional	America	883	Breast cancer, a history of PCOS, or missing information on PCOS	35–54	PM2.5 PM10 NO2	AMH	Women in the highest quartile of NO2 exposure, a traffic-related pollutant, had higher estimated AMH concentrations compared with the lowest quartile.	Age, education, BMI, and race/ethnicity.
Kim H, 2021	Retrospective	Asia	2,276	Women previously diagnosed with a chromosomal abnormality, having a history of unilateral or bilateral oophorectomy, and aged < 20 or > 49 years	36.6 ± 4.2	PM10 M2.5 NO_2_ CO SO_2_ O_3_	AMH	The study shows evidence indicating ambient PM10 concentration within a 1-month period is negatively associated with ovarian reserve in women with infertility; also, this negative association was additionally observed for PM2.5 within 1 month and 12 months in Seoul residents.	Age, BMI, season at the time of testing, previous smoking history.
Hood RB, 2021	Prospective	America	565	Women with incomplete scans, on Lupron (a Gonadotropin-releasing hormone antagonist), with PCOS, or missing air pollution exposure information	18–45	PM2.5	AFC	A higher exposure to PM2.5 was associated with lower AFC.	Age, BMI, smoking status, education, year, and season
Feng X, 2021	Retrospective	Asia	600	Women with COS for IVF treatment in this menstrual cycle before ovarian reserve assessment, women who did not reside in a registered residential address affiliated to Shanxi, PCOS, if one or both ovaries were difficult to visualize by using transvaginal ultrasonography scanning, hyperprolactinemia, hypothyroidism or immunologic diseases, Ovaries surgical treatment, chemotherapy, or radiotherapy, and RPL at least 3 times.	21–48	PM2.5 PM10 O_3_ NO_2_ CO SO_2_	AFC	In linear adjusted models, air pollutant SO_2_ is associated with lower AFC. Negative associations were observed between AFC and quartiles of NO_2_ levels compared with Q1.	Age, BMI, parity, and infertility diagnosis factors
Abareshi F, 2020	Cross-sectional	Asia	67	Women who did not have menses for 3 months or more, used infertility treatment, or had a history of polycystic ovarian syndrome, pelvic inflammatory disease, chemotherapy, or pelvic radiation.	32.3 ± 6.8	PM1 PM2.5 PM10	AMH	Inverse association between exposure to PM1, PM2.5, and AMH level. Direct association between exposure to PM1, PM2.5, and FSH but not significant in the fully adjusted models.	age, body mass index (BMI), education, regular menstrual cycle, parity, and smoking (yes/no).
La Marca A, 2020	Retrospective	Europe	1,463	exclusion of patients with severe chronic comorbidities reported in the reason for AMH analysis.	18–53	PM2.5 PM10 NO_2_	AMH	AMH levels were inversely related to environmental pollutants, such as PM10, PM2.5, and NO_2_. After subdividing the dataset into quartiles for PM10 and PM2.5, the influence of age on AMH serum levels was found to be stronger than that exerted by PM. For NO_2_ quartiles, higher AMH levels were observed in the third quartile compared to the fourth quartile, even after adjustment for age, indicating a more substantial influence of NO_2_ exposure on AMH serum levels.	Age
Ye X, 2020	Case–control	Asia		Eligibility criteria for POF cases: under 40 years of age at the first time of diagnosis; amenorrhea for at least 4 months; an increased FSH level > 25 IU/L on two occasions >4 weeks apart. Having known causes of POF (such as karyotypic abnormalities, ovarian surgery, autoimmune diseases, etc.) were excluded. Eligibility criteria for the controls included healthy women with regular menstrual cycles, without hormonal therapy in the last 6 months, and without endocrine system diseases.	33 ± 6	BaP	AMH	Among PAHs, BaP exhibited the strongest associations with these reproductive hormones in the logistic regression model. After adjustment for age, body mass index, educational levels, and household income, per one-unit increase in the log-transformed BaP (the most carcinogenic PAH congener) concentration was significantly correlated with a 2.191-fold increased risk of POF.	Age, body mass index, educational level, and household income.
Quraishi SM, 2019	Retrospective	America	7,463		34.9 ± 4.6	PM2.5 PM10 NO_2_	AFC	Women with DOR have high levels of exposure to PM2.5, PM10, and NO_2_ compared with those without DOR and who have low exposure.	Age, race, BMI, clinic location, and other infertility factors.
Gaskins AJ, 2019	Prospective	America	632	Incomplete scans, those done while the woman was on Lupron, those done on women with polycystic ovaries, repeated scans, and scans lacking complete exposure data.	18–45	PM2.5	AFC	Every 2 μg/m increase in estimated PM2.5 exposure was associated with a − 7.2% lower AFC adjusting for age, body mass index, smoking status, and year and season of the count. The association of PM2.5 with AFC was stronger among women with female factor infertility (−16.3% per 2 μg/m).	Age, BMI, smoking status, and year and season.

AMH, Anti-Müllerian hormone; BMI, Body Mass Index; RPL, Recurrent Pregnancy Losses; PCOS, Polycystic Ovary Syndrome; POR, poor ovarian reserve (< 4 oocytes retrieved); COH, Controlled ovarian hyperstimulation; Gn, Gonadotropins; FSH, Follicle-stimulating hormone; LH, luteinizing hormone; E2, Estradiol; AFC, Antral Follicle Count; DOR, Diminished Ovarian Reserve.

The study design of selected studies was found to be cross-sectional (2 of 15), retrospective cohort (10 of 15) (28–32, 34, 36, 38, 40–42), prospective cohort (2 of 15) (33, 39), and case–control (1 of 15) (37). The study population was distributed as follows: Asians (53%, eight studies), Americans (33%, five studies), and Europeans (14%, two studies).

Data extraction from the selected studies resulted in a total of 14 entries of environmental pollutants (PM, PM2.5, PM10, NO_2_, O_3_, CO, SO_2_, 1,3-butadiene, benzene, diesel PM, formaldehyde, methylene chloride, tetrachloroethylene, and benzo(a)pyrene). A majority of 65% of those resulting air pollutants, that is, PM1, PM2.5, PM10, NO_2_, O_3_, benzene, formaldehyde, SO_2_, and benzo(a)pyrene reported on a meaningful association with marker(s) of ovarian reserve. Indeed, no significant association resulted in CO, 1,3-butadiene, diesel PM, methylene chloride, and tetrachloroethylene about ovarian reserve. For ovarian reserve markers, the majority of the selected studies considered AMH (62%) as an outcome, followed by AFC (31%), and POR (7%).

Table 3 summarizes findings on different environmental pollutants associated with female ovarian reserve items. To focus on the evidence surrounding each environmental pollutant about ovarian reserve, the available retrieved literature will be elucidated as follows.

**Table 3:** Summary of findings on different environmental pollutants associated with ovarian reserve markers in childbearing females.

Exposure	Number of evidence based studies	Strength of association	Strength of evidence (GRADE)
PM1	2	PM1–AMH association by adjusted multivariable linear mixed effect model. For every 10 μg/m^3^ increment in PM1, the AMH changed by −8.8% (95% CI: from −12.1 to −5.3%), significant.	⊕ low
		In fully adjusted regression models, each one-IQR increase in PM1 was associated with −0.89 (95% confidence interval (CI): from−1.43 to −0.35, *p* ≤ 0.01) decrease in serum level of AMH.	
PM2.5	10	PM2.5–AMH association by adjusted multivariable linear mixed effect model. For every 10 μg/m^3^ increment in PM2.5, the AMH changed by −2.1% (95% CI: from −3.5 to −0.6%), significant.	⊕ ⊕ moderate
		Logistic regression models were employed to assess the association between quartiles of exposure to PM2.5 and the risk of POR. Women in the highest quartile of PM2.5 exposure during 6 months (OR: 1.44, 95% CI: 1.06–1.96) and 12 months (OR: 1.54, 95% CI: 1.10–2.14) before oocytes retrieval had a higher risk of POR than those in the lowest quartile, significant.	
		In multivariable models, an interquartile range (IQR) increase in 1- and 12-month average PM2.5 was associated with 3% (95% CI: from −0.07 to 0.00) and 10% (95% CI: from −0.18, to −0.01) lower AMH ratio, respectively.	
		In adjusted multivariable models, a 2 μg/m^3^ increase in average PM2.5 exposure was associated with a 6.2% (95% CI: from −11.8 to −0.3) lower AFC.	
		In fully adjusted models, each one-IQR increase in PM2.5 was associated with a − 1.11 (95% CI: from −1.67 to −0.55, *p* ≤ 0.01) decrease in serum level of AMH.	
		In adjusted models, AMH levels were inversely related to PM2.5 (*ρ* = −0.062, *p* = 0.021).	
		Women with DOR had high levels of exposure to PM2.5 (*p* = 0.003) compared with those without DOR and who had low exposure.	
		Every 2-μg/m increase in estimated PM2.5 exposure was associated with a − 7.2% (95% CI: from −10.4 to −3.8%) lower antral follicle count.	
		In the adjusted multivariate model, a significant association between ovarian reserve parameters and air pollution was observed in the fourth quartile of PM2.5 exposure for AFC (*p* = 0.044) and AMH (*p* = 0.032) compared with the first quartile when exposure was treated as a categorical variable.	
		When PM2.5 exposure levels were equal to the 50th percentile (32.6–42.3 μg/m^3^) or more, monotonically decreased AMH levels and increased risks of low AMH were seen with increasing PM2.5 concentrations during W1 and W4 (*p* < 0.05).	
PM10	5	PM10–AMH association by adjusted multivariable linear mixed effect model. For every 10 μg/m^3^ increment in PM10, the AMH changed by −1.9% (95% CI: from −3.3 to −0.5%), significant.	⊕ ⊕ moderate
		Logistic regression models were employed to assess the association between quartiles of exposure to PM10 and the risk of POR. Women in the third quartile of PM10 exposure for 3 months (OR: 0.82, CI: 95%: 0.70–0.97) and 6 months (OR: 0.78, 95% CI: 0.66–0.91) before oocytes retrieval had a higher risk of POR compared with those in the lowest quartile, significant.	
		In multivariable models, an IQR increase in 1-month average PM10 was associated with a decrease (β-coefficient = −0.06, 95% CI: from −0.11 to 0.00, Table 2) in the AMH ratio.	
		In adjusted models, AMH levels were inversely related to PM10 (*ρ* = −0.088, *p* = 0.001), significant.	
		Women with DOR had high levels of exposure to PM10 (*p* = 0.01) compared with those without DOR and who had low exposure.	
Benzene	1	Benzene–AMH association by adjusted linear models. A negative association of AMH with benzene (percentage reduction in AMH per IQR increase = 5.5, 95% CI: 1.0–9.8), significant.	⊕ very low
Formaldehyde	1	Formaldehyde–AMH association by adjusted linear models. A negative association of AMH with formaldehyde (percentage reduction in AMH per IQR increase = 6.1, 95% CI: 1.6–10), significant.	⊕ very low
O_3_	3	O_3_–AMH association by adjusted multivariable linear mixed effect model. For every 10 μg/m^3^ increment in O_3_, the AMH changed by −4.5 (95% CI: from −7.1 to −1.9), significant	⊕ low
		Women in the third quartile of O_3_ exposure for 3 months (OR: 1.19, 95% CI: 1.00–1.42) and 12 months (OR: 1.28, 95% CI: 1.08–53) before oocytes retrieval had a higher risk of POR than those in the lowest quartile, significant.	
		In linear adjusted models, each 10 μg/m^3^ increase in ozone was associated with 2.34% (0.68, 3.97%), 2.08% (0.10, 4.01%), 4.20% (1.67, 6.67%), and 8.91% (5.79, 11.93%) decreased AMH levels during W1–W4.	
SO_2_	3	Logistic regression models were employed to assess the association between quartiles of exposure to SO_2_ and the risk of POR. Women in the third quartile of SO_2_ exposure for 6 months (OR: 2.10, 95% CI: 1.67–2.64) and 12 months (OR: 2.53, 95% CI: 2.01–3.19) before oocytes retrieval had a higher risk of PORthan those in the lowest quartile, significant.	⊕ low
		In linear adjusted models, every 10 μg/m^3^ increase in SO_2_ concentration level during the entire development stage of antral follicle was associated with a − 0.01 change in AFC (95% CI: from −0.016 to −0.002), significant.	
		In adjusted multivariate model, SO2 concentrations significantly decrease AFC (*p* = 0.038).	
NO_2_	5	NO_2_–AMH association by adjusted multivariable linear mixed effect model. For every 10 μg/m^3^ increment in NO_2_, the AMH changed by −4.5 (95% CI: from −7.1 to −1.9), significant.	⊕ ⊕ moderate
		Multivariable-adjusted linear regression to estimate the percent change in AMH in relation to ambient residential NO_2_ (quartile exposure). Women in the highest quartile of NO_2_ exposure had higher estimated AMH concentrations (Q4 vs. Q1, 42.9%; 95% CI: from −3.4 to 111.4) compared with the lowest quartile, not significant.	
		In linear adjusted models, negative associations were observed between AFC and quartiles of NO_2_ levels: Q2 (−0.138 change, 95% CI: from −0.198 to −0.078), Q3 (−0.058 change, 95% CI: from −0.170 to 0) and Q4 (−0.068 change, 95% CI: from −0.127 to −0.009) compared with Q1, significant.	
		In adjusted models, AMH levels were inversely related to NO_2_ (*ρ* = −0.111, *p* < 0.001), significant.	
		Women with DOR had high levels of exposure to NO_2_ (*p* < 0.001) compared with those without DOR and who had low exposure.	
BaP	1	In adjusted logistic regression models, per one-unit increase in the log-transformed BaP concentration was significantly correlated with a 2.191-fold increased risk of POF (OR: 2.191, 95% CI: 1.6–2.9, *p* < 0.05).	⊕ very low

### PM2.5 and ovarian reserve

For the pollutant PM2.5, 10 studies (28, 30, 32, 33, 35, 36, 38, 39, 41, 42) were retrieved, providing moderate strength of evidence. Six of these studies considered AMH as an outcome: Pang and colleagues (28) indicated that for every increase of 10 μg/m^3^ in ambient PM2.5, AMH changed by −2.1% (95% confidence interval [CI]: from −3.5 to −0.6); Kim and colleagues (32) indicated that an increase in the interquartile range (IQR) in mean PM2.5 at 1 and 12 months was associated with a 3% (95% CI: from −0.07 to 0.00) and 10% (95% CI: from −0.18 to −0.01) (28, 30, 32, 33, 35, 36, 38, 39); Abareshi and colleagues (35) showed that each increase of an IQR of ambient PM2.5 was associated with a decrease in serum AMH level of −1.11 (95% CI: from −1.67 to −0.55); La Marca and colleagues (36) showed AMH levels weakly inversely correlated with PM2.5 (*ρ* = −0.062, *p* = 0.021) in the adjusted models; Wieczorek and colleagues (42) found a negative association between PM2.5 exposure and AMH and AFC levels; and finally, Liu and colleagues (41) demonstrated a significant drop in AMH levels and increased risk of low AMH associated with PM2.5 exposure during the secondary and antral phases and 1 year before the measurement, even below the current Chinese air quality standard on PM2.5 concentrations (75 μg/m^3^). The other four studies considered AFC or decreased/poor ovarian reserve (DOR/POR) as an outcome: Wu and colleagues (30) used logistic regression models to assess the association between quartiles of PM2.5 exposure and POR risk finding that women in the highest quartile of PM2.5 exposure during 6 months (OR: 1.44, 95% CI: 1.06–1.96) and 12 months (OR: 1.54, 95% CI: 1.10–2.14) before oocytes retrieval had a higher risk of POR than those in the lowest quartile; Hood and colleagues (33) demonstrated a 2 μg/m^3^ increase in mean PM2.5 exposure to be associated with an AFC reduction of −6.2% per 2 μg/m^3^ (1 standard deviation (SD) increase; 95% CI: from −11.8 to −0.3) in multivariable adjusted models; Quraishi and colleagues (38) showed that women with DOR had high levels of PM2.5 exposure (*p* = 0.003) compared with those without DOR and with low exposure; finally, Gaskins and colleagues (39) showed that each 2 μg/m increase in estimated PM2.5 exposure was associated with a reduction of −7.2% (95% CI: = from −10.4 to −3.8%) lower AFC count.

### PM10 and ovarian reserve

For the pollutant PM10, a total of five studies were retrieved (28, 30, 32, 36, 38), providing moderate strength of evidence. Three of these studies considered AMH as an outcome: Kim and colleagues (32) found an inverse PM10–AMH association by multivariable linear mixed-effects adjusted model. For each 10 μg/m^3^ increase in PM10, AMH varied by −1.9% significantly (95% CI: from −3.3 to −0.5%); Pang and colleagues (28) developed multivariable models showing an increase in the IQR of mean PM10 at 1 month was associated with a decrease (*β*-coefficient = −0.06, 95% CI: from −0.11 to 0.00) of AMH ratio; Quraishi and colleagues (38) found AMH levels inversely correlated with PM10 (*ρ* = −0.088, *p* = 0.001), and the findings were significant for women with a reduced ovarian reserve in adjusted models. The other two studies considered POR and DOR as an outcome: La Marca and colleagues (36) used logistic regression models to assess the association between PM10 exposure quartiles and the risk of POR. Women in the third quartile of PM10 exposure for 3 months (OR: 0.82, 95% CI: 0.70–0.97) and 6 months (OR: 0.78, 95% CI: 0.66–0.91) before oocytes retrieval had a higher risk of POR than those in the lowest quartile; Wu and colleagues (30) showed that women with decreased ovarian reserve (DOR) had high levels of PM10 exposure (*p* = 0.01) compared with those without DOR and with low exposure.

### NO_2_ and ovarian reserve

For the pollutant NO_2_, a total of five studies were retrieved (28, 31, 34, 36, 38), providing moderate strength of evidence. Three of these studies considered AMH as an outcome: Feng and colleagues (34) reported a significant inverse NO_2−_AMH association by a multivariable linear mixed-effect adjusted model. Here, for each 10 μg/m^3^ increase in NO_2_, AMH changed by −4.5% (95% CI: from −7.1 to −1.9) significantly; Gregoire and colleagues (31) performed a multivariable-adjusted linear regression to estimate the percent change in AMH in relation to residential ambient NO_2_ (exposure quartile) and found that women in the highest quartile of NO_2_ exposure had higher estimated AMH concentrations (Q4 vs. Q1, 42.9%; 95% CI: from −3.4 to 111.4) than the lowest quartile; however, the data lacked statistical significance; Pang and colleagues (28) demonstrated that in the adjusted models, AMH levels were inversely, statistically related to NO_2_ (*ρ* = −0.111, *p* < 0.001). The other two studies considered AFC and DOR as an outcome: La Marca and colleagues (36) performed adjusted linear models observing negative, statistically significant associations between AFC and quartiles of NO_2_ levels: Q2 (−0.138 change, 95% CI: from −0.198 to −0.078), Q3 (−0.058 change, 95% CI: from −0.170 to 0) and Q4 (−0.068 change, 95% CI: from −0.127 to −0.009) compared with Q1; Quraishi and colleagues (38) showed that women with DOR had high levels of NO_2_ exposure (*p* < 0.001) compared with those without DOR and with low exposure, and the difference was statistically significant.

### PM1 and ovarian reserve

For the pollutant PM1, only two studies were retrieved (28, 35), providing an overall low strength of evidence. Abareshi and colleagues (35) studied the PM1 − AMH association by multivariable linear mixed-effects adjusted model, finding that for each 10 μg/m^3^ increase in PM1, AMH varied significantly by −8.8% (95% CI: from −12.1 to −5.3%). Pang and colleagues (28) implemented fully adjusted regression models, finding that each IQR increase in ambient PM1 was associated with a − 0.89 (95% CI: from −1.43 to −0.35, *p* ≤ 0.01) decrease in serum AMH level.

### SO_2_ and ovarian reserve

For the pollutant SO_2_, only three studies were retrieved (30, 34), providing an overall low strength of evidence. Feng and colleagues (34) employed logistic regression models to assess the association between quartiles of SO_2_ exposure and the risk of POR, finding that women in the third quartile of SO_2_ exposure during 6 months (OR: 2.10, 95% CI: 1.67–2.64) and 12 months (OR: 2.53, 95% CI: from 2.01 to 3.19) before oocytes retrieval had a higher risk of POR than those in the lowest quartile. Wieczorek and colleagues (42), in adjusted multivariate models, found SO_2_ concentrations significantly decreased AFC (*p* = 0.038). Wu and colleagues (2) employed adjusted linear models showing a 10 μg/m^3^ increase in SO_2_ concentration level during the entire antral follicle development phase to be statistically associated with a − 0.01 change in AFC (95% CI: form −0.016 to −0.002).

### O_3_ and ovarian reserve

For the pollutant O_3_, only three studies were retrieved (28, 30), providing an overall low strength of evidence. Pang and colleagues (28) studied the O_3−_AMH association by multivariable linear mixed-effect adjusted model, finding that for each 10 μg/m^3^ increase in O_3_, AMH varied significantly by −4.5 (95% CI, from −7.1 to −1.9). In adjusted linear models, Liu and colleagues (40) showed that each 10 μg/m^3^ increase in ozone was associated with a decrease in AMH levels of 2.34% (0.68, 3.97%), 2.08% (0.10, 4.01%), 4.20% (1.67, 6.67%), and 8.91% (5.79, 11.93%) during W1–W4. Wu and colleagues (2) showed that women in the third quartile of O_3_ exposure during 3 months (OR: 1.19, 95% CI: 1.00–1.42) and 12 months (OR: 1.28, 95% CI: 1.08–1.53) before oocytes retrieval had a higher risk of POR than those in the lowest quartile.

### Benzene and ovarian reserve

For the pollutant benzene, only one report was retrieved (29), providing an overall very low strength of evidence. Li and colleagues studied the benzene–AMH association using adjusted linear models, finding a negative, significant association of AMH with benzene [percent reduction in AMH per increase in interquartile range (IQR) = 5.5, 95% CI: 1.0–9.8].

### Formaldehyde and ovarian reserve

For the pollutant formaldehyde, only one report was retrieved (29), providing an overall very low strength of evidence. Li and colleagues studied the formaldehyde–AMH association using adjusted linear models and found a negative and statistically significant association of AMH with formaldehyde (percent reduction in AMH per increase in the IQR = 6.1, 95% CI = 1.6–10).

### Benzo(a)pyrene and ovarian reserve

For the pollutant benzo(a)pyrene (BaP), only one report was retrieved (37), providing an overall very low strength of evidence. Ye and colleagues ran adjusted logistic regression models, finding each one-unit increase in log-transformed BaP concentration to be significantly related to a 2.191-fold increased risk of premature ovarian failure (POF; OR: 2.191, 95% CI: 1.6–2.9, *p* < 0.05).

### Quality assessment and risk of bias

**Figure 2:**
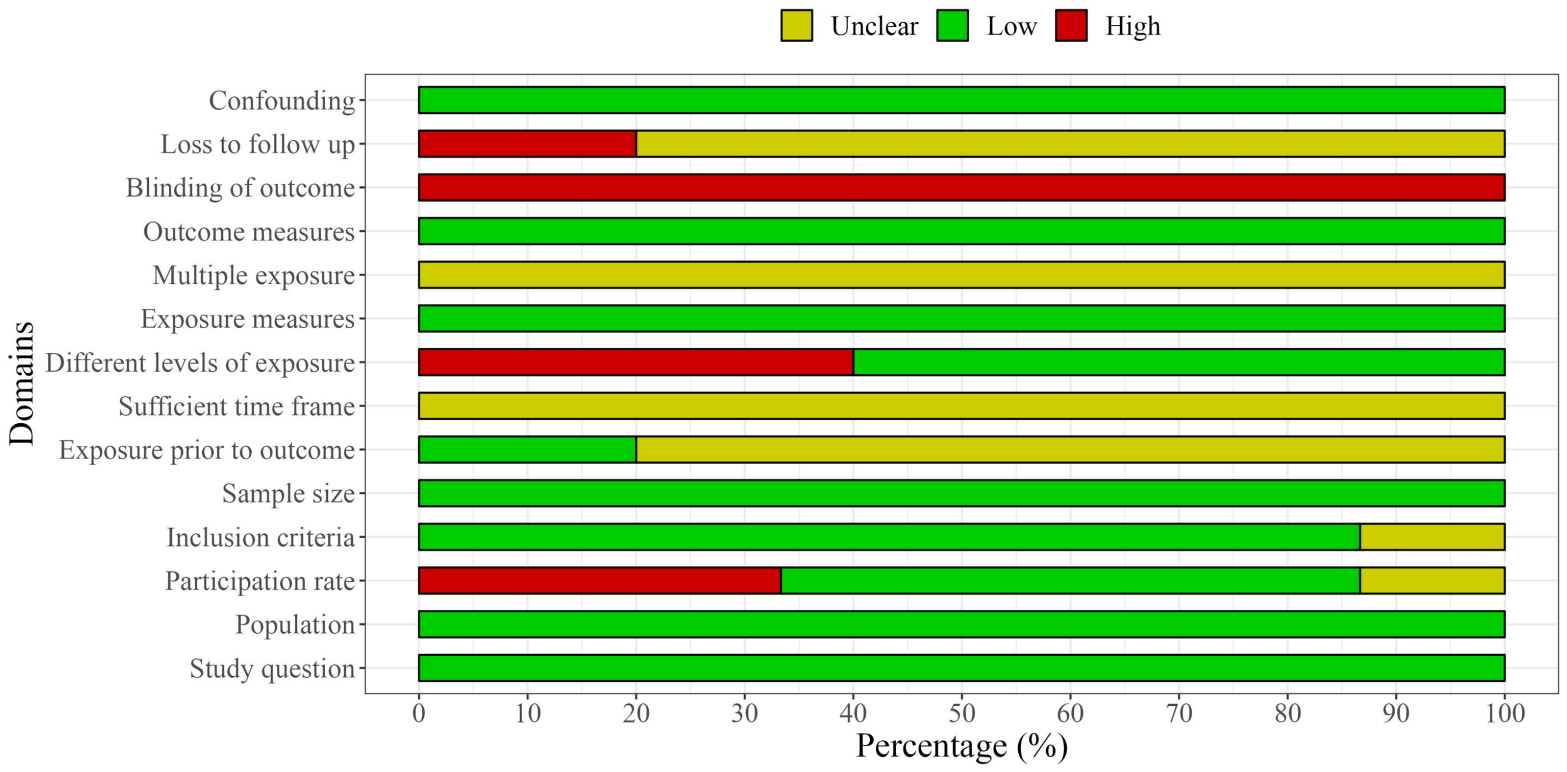
Quality assessment plot across domains.

**Figure 3:**
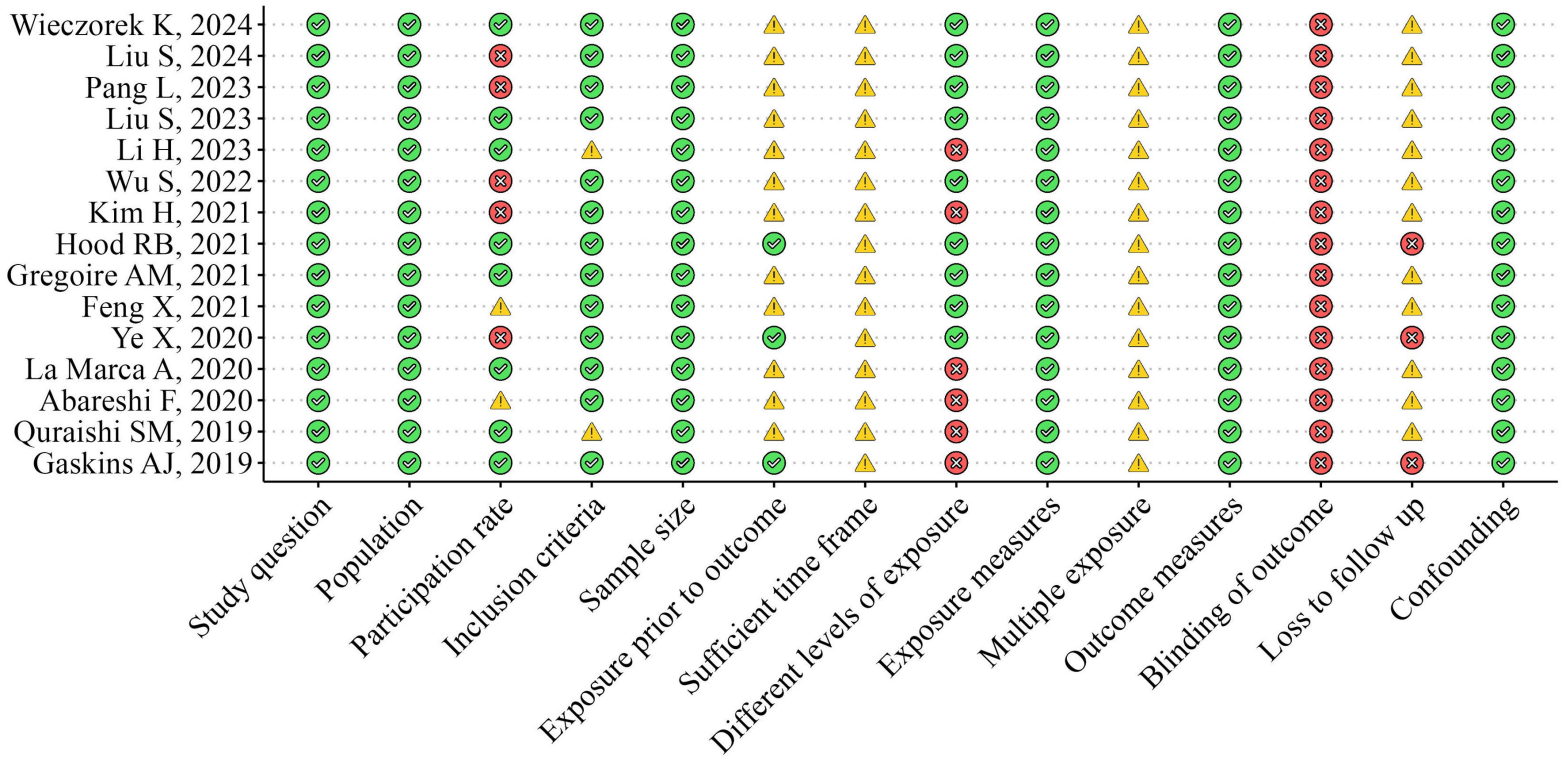
Risk of bias assessment across selected studies.

The methodological quality of the included studies was independently assessed by two researchers (RZ and FC) using the National Institutes of Health Quality Assessment Toolkits for Observational Cohort and Cross-Sectional Studies (25, 26) (Figures 2, 3). The overall methodological quality of the selected studies was rated moderate across the 14 toolkit domains. In particular, the risk was rated low for domains such as study question, population, exposure measures, outcome measures, sample size, and confounding factors across studies. Some concerns arose in some studies for domains such as inclusion criteria, participation rate, and multiple exposure. In contrast, a medium to high risk of bias was found for the domain participation rate, blinding of the outcome, and loss to follow-up across selected studies.

## Discussion

This review aimed to systematically explore the association between major environmental pollutants and markers of ovarian reserve as proxies of female fertility. After retrieving 15 original reports from the literature screening process, we found a cluster of exposure items reporting on PM1, PM2.5, PM10, NO_2_, O_3_, SO_2_, CO, PAHs, 1,3-butadiene, benzene, diesel PM, formaldehyde, methylene chloride, and tetrachloroethylene as environmental pollutants in relation to ovarian reserve markers as AMH, AFC, and indices of poor or reduced ovarian reserve. The main findings showed a higher body of evidence for the environmental pollutants PM2.5, PM10, and NO_2_, while a low body of evidence for PM1, O_3_, SO_3_, and a very low body of evidence for benzene, formaldehyde, and benzo(a)pyrene, yet consistently showing significant inverse association data.

Although the mechanisms underlying the adverse health effects of exposure to air pollution have not yet been established, inflammation and oxidative stress have been suggested to be the key pathways. Indeed, folliculogenesis has been described to be impaired by increased oxidative stress and cell apoptosis induced by ambient polluted air (43).

For the inflammatory pathway, it has been reported that PM2.5 exposure can support the enhancement of inflammatory fluid markers, as indicated by changes in IL-6 and TNF-levels (44), as well as morphological changes in ovarian tissue, such as mitochondrial structural changes, vascular congestion, and hemorrhage, triggered by the inflammation itself (39). Therefore, this response might result in ovarian damage and reduced fertility. Along these lines, findings on animal models showed that IL-6 and TNF-*α* concentrations and the number of apoptotic cells were increased in ovarian tissue and histological structures of the ovary showing signs of hemorrhage and vascular congestion in mice exposed to PM2.5 compared with the control group (22).

For oxidative stress, reactive oxygen species (ROS) and mitochondrial DNA (mtDNA) have been shown to impact cellular aging in the human body, including in the female reproductive tract (45). Some studies have suggested that an excess of ROS May hurt ovarian aging. ROS are highly reactive oxygen-containing compounds, such as superoxide anions, hydrogen peroxide, and hydroxyl radicals. ROS are formed endogenously by oxygen metabolism during cellular processes. Usually, cells can eliminate excess ROS; however, when produced in excess, these compounds cause oxidative stress and cellular damage. High concentrations of ROS in cells lead to mitochondrial and nuclear DNA damage and apoptosis. These types of damage have been shown to affect ovarian follicle development and ovulation negatively.

Further explaining the findings, three other paths have been described by the scientific community so far, which include vitamin D3 metabolism, vitamin A metabolism, and bile acid biosynthesis. Indeed, vitamins D and A have long been implicated in human reproduction. Vitamin D signaling is directly involved in the expression of AMH, which is produced by ovarian granulosa cells and is known for its role in regulating follicular recruitment and selection. Therefore, vitamin D deficiency in females May contribute to impaired ovarian physiology through altered AMH signaling (46). Given that enzymes known to be involved in retinoid synthesis are found in the ovary, it is plausible that vitamin A deficiency May lead to deterioration in oocyte quality. Emerging evidence also suggests that air pollution May directly (through reduced ultraviolet B [UVB] exposure) and indirectly (through reduced time spent outdoors) decrease skin production of vitamin D3 (47) and reduce levels of the vitamin A precursor, *β*-carotene (a potent antioxidant), in the body (48).

Last, as little evidence has linked exposure to PAHs to ovarian reserve, it is useful to point out that PAHs are ubiquitous environmental pollutants worldwide and generated mainly during incomplete combustion of organic materials, including anthropogenic combustion sources (vehicle emissions, cigarette smoke, waste incineration, and so on) and natural combustion sources (volcanic activities, forest fires, etc.) (49, 50). Inhalation, ingestion, and skin contact are the main routes of exposure to PAHs. BaP, as the most carcinogenic PAH congener, has been described to retard follicular development in the ovary and decrease follicle viability, probably through activation of Aryl hydrocarbon Receptor (AhR) signaling (51). Here, reports on animal models also found that exposure to traffic-related air pollution correlated with a reduction in the number of antral follicles (23, 52, 53), yet further human research is needed to fill the gap.

### Limitations

First, the ascertainment of exposure was heterogeneous among the studies. Most studies assessed air quality using a specific air monitoring station, while others estimated exposure based on proximity to the potential source. In addition, the reference levels of each pollutant could vary between studies. These factors, together with the small number of articles found, make a quantitative approach to this problem difficult.

## Conclusion

Increased exposure to air pollutants might be associated with reduced female ovarian reserve, and while the evidence is more substantial for pollutants such as PM2.5, PM10, and NO_2_, more evidence is needed to allow conclusions about causality to be drawn. In light of these findings, global action is required for all significant modern pollutants. Global efforts May act synergistically with other international environmental policy programs to avoid any of the risks associated with this topic, such as birth rate cuts or the use of assisted reproductive technologies. A rapid and large-scale transition from all fossil fuels to clean, renewable energy is a win-win strategy to prevent pollution while mitigating climate change, thereby achieving a double benefit for the planet’s health.

## Data Availability

The raw data supporting the conclusions of this article will be made available by the authors, without undue reservation.

## Footnotes

^1^ https://arxiv.org/in

^2^ http://www.opengrey.eu/
