# Supplementary figures and images for: Air pollutants and ovarian reserve: a systematic review of the evidence
Source: Front Public Health. 2024 Sep 23;12:1425876. doi: 10.3389/fpubh.2024.1425876 (PMC11457886; doi:10.3389/fpubh.2024.1425876)

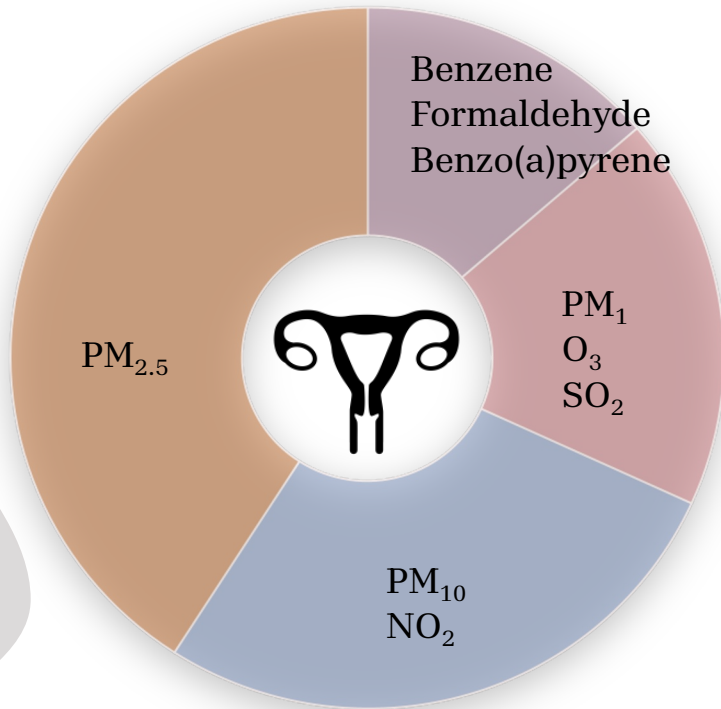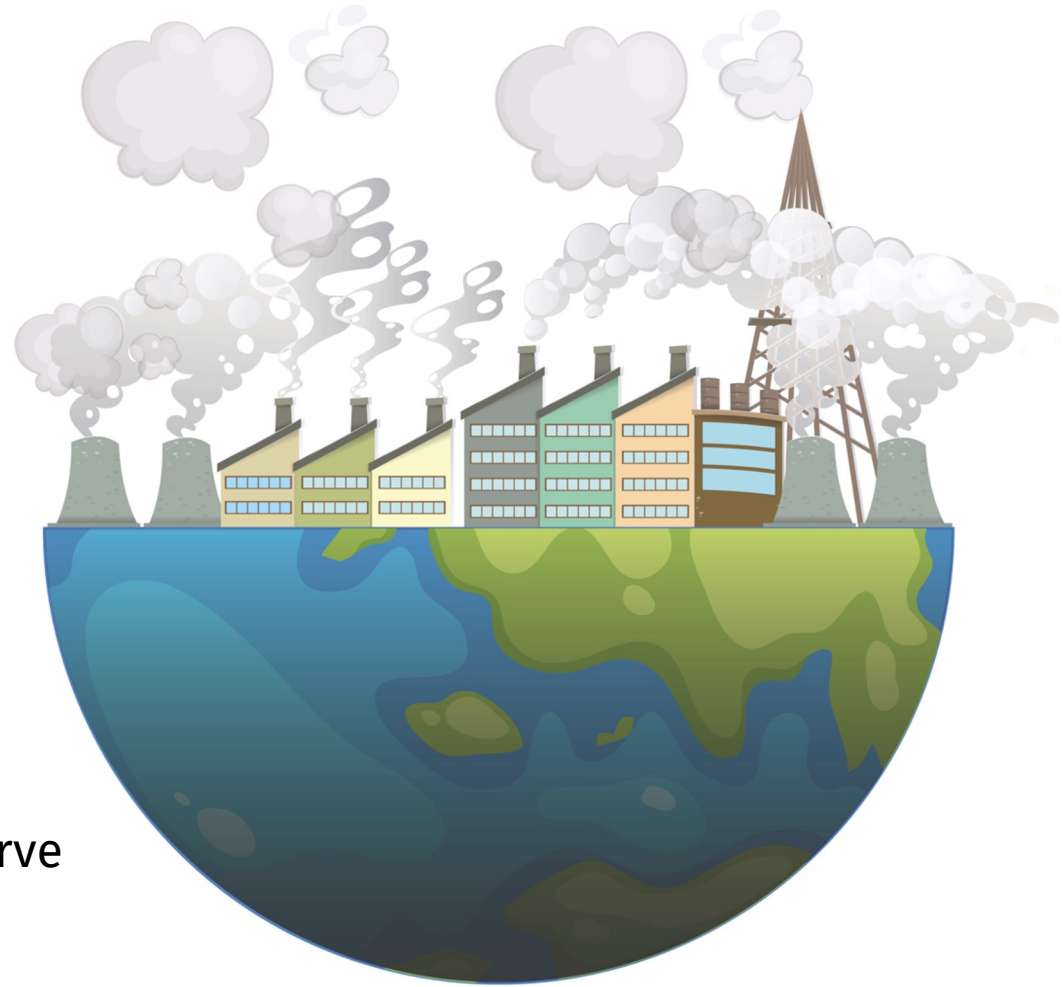

High evidence indicates PM<sub>2.5</sub> lowers ovarian reserve

Supplement: Supplementary file 1 [file Data_Sheet_1.PDF]
